# A process evaluation of a stroke-specific follow-up care model for stroke patients and caregivers; a longitudinal study

**DOI:** 10.1186/s12912-014-0052-8

**Published:** 2015-01-16

**Authors:** Manon Fens, George Beusmans, Martien Limburg, Liesbeth van Hoef, Jolanda van Haastregt, Job Metsemakers, Caroline van Heugten

**Affiliations:** Department of Patient & Care, Maastricht University Medical Centre+, Maastricht, The Netherlands; CAPHRI School for Public Health and Primary Care, Maastricht University, Maastricht, The Netherlands; Department of General Practice, Maastricht University Medical Centre+, Maastricht, The Netherlands; Department of Neurology, Flevo Hospital, Almere, The Netherlands; Department of Health Services Research, Maastricht University, Maastricht, The Netherlands; Department of Psychiatry and Neuropsychology, School for Mental Health and Neuroscience, Maastricht University, PO Box 616, 6200 MD Maastricht, The Netherlands; Department of Neuropsychology and Psychopharmacology, Faculty of Psychology and Neurosciences, Maastricht University, Maastricht, The Netherlands

**Keywords:** Follow-up care, Nurses, Process assessment, Stroke

## Abstract

**Background:**

There is a need for follow-up care after stroke, but there is no consensus about the way to organise it. An intervention providing follow-up care for stroke patients and caregivers showed favourable effects on the level of social activities, but no other effects were found. The intervention consists of a maximum of five home visits to patients and caregivers during a period of 18 months post-discharge. The home visits are conducted by a stroke care coordinator (SCC) using a structured assessment tool. The objective of this study was to examine process-related factors that could have influenced the effectiveness of the intervention.

**Methods:**

77 stroke patients, 59 caregivers and 4 SCCs participated in the study. Data on the organisational characteristics of and the satisfaction with the intervention were collected by means of structured assessments, interviews and self-administered questionnaires at 1, 6, 12 and 18 months of follow-up. The intervention was provided between April 2008 and June 2011.

**Results:**

Patients received an average of 3.8 home visits (SD 1.4) and 55% of them had a follow-up period of a maximum of 18 months. There were 1074 problems identified and the SCCs initiated 363 follow-up care and referral options. Stroke patients and caregivers were very satisfied with the intervention. The SCCs were satisfied with the assessment tool, but would like to see a structured referral system.

**Conclusions:**

The intervention was only partially performed in accordance with the protocol and was positively evaluated by patients, caregivers and SCCs. It is recommended to add a structured referral system to the intervention.

## Background

Many stroke patients experience motor, cognitive and psycho-emotional deficits or problems with daily activities and social participation [1-3], which are often persistent. Their caregivers are also affected by the consequences of stroke and often experience psychological and emotional problems [4,5]. Stroke patients as well as their caregivers have a need for long-term care [6], but this long-term care is complex because many functions can be affected and many health care professionals may be involved.

Previous studies have evaluated different long-term care models for stroke patients, but only a few have shown significant favourable effects [7,8]. Allen and colleagues (2002) evaluated a standardised assessment for stroke patients one month after being discharged home, followed by an individual care plan developed by a multidisciplinary team [7]. They found that quality of life had improved after three months. Another study compared the effect of an intensive face-to-face therapy with that of a less intensive face-to-face therapy for patients living at home after hospitalisation, both of which were provided by a multidisciplinary team [8]. The intensive therapy proved more effective in terms of quality of life. The care models that were reported to be effective were however, very heterogeneous in terms of type of care, professionals involved, and duration and intensity of care, which makes comparisons difficult and extracting effective elements impossible [9]. There does not seem to be consensus about the best way to organise follow-up care for stroke patients who are discharged home. Based on previous research [6,10] and practice-based evidence, we suggest that long-term care should be available for all stroke patients and that they should be monitored regularly for at least one year after hospitalisation or inpatient rehabilitation, as they can experience persistent long-term problems. We therefore developed an intervention for stroke patients being discharged home from hospitalisation or inpatient rehabilitation, and their caregivers. The intervention consists of five home visits by a stroke care coordinator (SCC) employed by the home care services, using a structured assessment procedure to assess stroke-related problems and to offer adequate follow-up care. To design this structured assessment procedure, a comprehensive assessment tool was needed, however there were no adequate tools available for use in clinical practice. Therefore we have developed the Assessment tool for long-term Consequences After Stroke (ACAS) [11]. This intervention (including the ACAS) was implemented in the Maastricht area, the Netherlands, and compared with regular care in a control area (Eindhoven, the Netherlands) to evaluate its effectiveness in a non-randomised controlled trial design. Although the results showed that the intervention had a favourable effect on the levels of social activities of stroke patients, no significant favourable effects between the intervention and control group were found regarding quality of life, activities of daily life, depression and anxiety, or caregiver strain [12]. Alongside the effect evaluation study, we performed a process evaluation to assess whether all stroke patients had received the intervention, whether the intervention was carried out in accordance with the protocol and whether the care provided was feasible and evaluated favourably by patients, caregivers and care professionals.

### Aim

The aim of this study was to examine process-related factors that may have influenced the effectiveness of the intervention. This evaluation was divided into two parts. The first part focused on the availability of the intervention for all stroke patients and evaluated (1) whether each stroke patient had been referred to the SCC to receive the intervention after being discharged home. The second part of the evaluation concentrated on the intervention itself and evaluated (2) to what extent the intervention was performed in accordance with the protocol, (3) to what extent the assessment resulted in follow-up care, (4) what the opinion of the patients and caregivers about the intervention was, and (5) what the opinion of the SCCs about the intervention was.

## Methods

### Design

The study was conducted alongside the study into the effectiveness of the intervention and had a longitudinal mixed methods design and focused on both quantitative and qualitative outcomes.

### Participants

This process evaluation focused on stroke patients and their caregivers in the Maastricht area who participated in the intervention group of the trial [12]. Patients were included if they had been diagnosed with a stroke, were aged 50 years or older, and were living in the community in the catchment area of the home care services performing the intervention. The caregivers were included if they were 18 years or older and were the primary caregiver of the included stroke patient. All four SCCs of the home care services providing the care were asked to participate in the process evaluation.

### Intervention

All stroke patients were referred to the SCC of home care services after being discharged home from hospital or inpatient rehabilitation. The SCCs were nurses, who were specialised in stroke and in long-term care after stroke. The intervention consists of five home visits to stroke patients and their caregivers by the SCC over a period of 18 months. The first home visit had to be performed within one month after discharge, followed by visits at 3, 6, 12 and 18 months after discharge. During each home visit, the SCC used the ACAS to identify problems within the broad spectrum of stroke-related problems. This assessment tool was developed in order to be used in the intervention [11]. This assessment tool consists of 17 items relating to the 12 domains of activities of daily life (ADL), instrumental activities of daily life (IADL), social activities, cognition, communication, psycho-emotional status, fatigue, secondary prevention, medical consumption, medical condition, caregiver strain and provision of information. Each domain has a hierarchical structure and starts with a brief question to explore whether the patient is experiencing a problem. If no problems are present, no specifications are required. If a problem is present, that problem can be explored further by means of validated measurement instruments [13-18]. The assessment provides a broad overview of the patient’s needs, so follow-up care can subsequently be provided. The SCC can contact the patient’s general practitioner (GP) for advice about referral options. They can also consult a multidisciplinary team for advice about patients experiencing complex problems. This team, consisting of a nursing home physician, physiotherapist, speech therapist, occupational therapist and rehabilitation physician, was specifically established for this intervention. The intervention was conducted between April 2008 and June 2011.

### Data collection

Based on the framework of Saunders and colleagues [19], we assessed the following process outcomes: availability of the intervention (reach); performance of the intervention (fidelity/dose delivered); follow-up care based on assessment (dose received, exposure); opinion of the patients and caregivers and opinion of the stroke care coordinators (dose received, satisfaction). The methods used to assess these process outcomes are described below.

#### Part 1

##### Availability of the intervention

The registration systems of the hospital, inpatient rehabilitation setting and home care services were used to assess whether all stroke patients were indeed referred to home care services after being discharged home. The researcher checked how many patients were discharged home after hospitalisation or inpatient rehabilitation during the study period, and how many of these patients were referred to the home care services to receive the intervention.

#### Part 2

##### Performance of the intervention

In order to assess whether the home visits and follow-up period was implemented according to our suggested intervention, the following data was recorded by the SCCs for each participant on the ACAS form: the timing of the home visits, the number of home visits, and the duration of the follow-up period. Furthermore, the SCCs registered whether they used the additional validated measurement instruments during the assessments.

##### Follow-up care based on assessment

The number and type of problems reported by the patients and caregivers during the home visits were recorded by the SCCs on a structured assessment form. On this form, the SCCs also registered the follow-up care they had initiated for each patient.

##### Opinions of patients and caregivers

Patients’ and caregivers’ satisfaction with care provided was measured using the Satisfaction with Stroke-Care questionnaire (SASC-19). The researcher interviewed the participants and caregivers one month after the patient had been discharged home (T0), and subsequently every six months after the baseline measurement for the entire 18 month follow-up period (i.e. T6, T12 and T18). These measurements were scheduled to take place shortly after a home visit by the SCC when possible. Patients were instructed to indicate how satisfied they were with the care that they were receiving. Caregivers were also asked to complete the SASC-19 and were also instructed to indicate their satisfaction with the care that they were receiving. The SASC-19 consists of two parts, with a total of 19 items: part one contains items about hospital care, while part two contains items about care after hospitalisation. Each item has a 4-point scale, ranging from 0 (totally disagree) to 3 (totally agree). For the purpose of our study, four items of part one were changed regarding the care referred to by the items (from hospital care to care by the SCCs; items 1, 2, 3 and 5). In part two, inpatient rehabilitation was added to hospital care for items 9, 13, 15, 16 and 17.

The way the intervention was organised was evaluated by presenting patients and caregivers with a self-administered questionnaire after 18 months. The patients and caregivers were both asked to answer the same questions, consisting of 16 multiple-choice questions (agree-disagree or too short-too long), two open-ended questions (likes and dislikes about the care) and one question asking to rate the way the intervention was organised (1 (bad) -10 (excellent)). The multiple-choice questions addressed aspects such as the timing of the first home visit, the expertise of the SCC, the use of validated questionnaires by the SCC, the number of home visits, the duration of the home visits and the location of the home visits. The researcher asked the patients and caregivers to return the questionnaires by post. If the questionnaire was not returned, the researcher called the patient and caregiver to remind them.

##### Opinion of the stroke care coordinators

The SCCs received a questionnaire, which was developed for our study purposes, at the end of the study period to assess their opinion on the organizational characteristics of the intervention that they provided. The questionnaire consisted of four parts; part one focused on the elements of the intervention (7 multiple choice items); part two addressed aspects of the use of the assessment tool (9 items; one rating on a scale from 0-10, four multiple choice and four open questions); part three consisted of two multiple choice items about the working conditions; and part four addressed the multidisciplinary collaboration between the health care professionals (6 items; two ratings on a scale from 0-10, two multiple choice and two open questions).

### Ethical considerations

The medical ethics committee of the Maastricht University Medical Centre approved this study and all patients and caregivers gave informed consent.

### Data analysis

Means and standard deviations or percentages were used to describe participants’ and disease characteristics. The quantitative data about the structured assessments (such as the number of home visits and the duration of the follow-up period), as reported by the SCCs, are presented as means, standard deviations, frequencies and percentages. The data from the assessments (such as the type of problem and the follow-up care initiated) are presented as frequencies and percentages. The sum scores of the SASC-19 could not be interpreted, because many items were not applicable and could not be scored. Therefore we used the average degree of satisfaction reported by each participant, to obtain an estimate of the satisfaction with care. The average degree of satisfaction was calculated for each patient and caregiver by dividing the sum score by the number of items which were scored (i.e. a relative score; we considered a score between 0 and 1.5 as total to moderate dissatisfaction and a score between 1.6 and 3 as moderate to total satisfaction). SPSS (version 18) was used for all statistical analyses.

### Validity and reliability

The ACAS tool administrated by the SCCs has good content and criterion validity and has been rated as feasible for use in health care [11]. The SASC-19 has good validity [20]. The questionnaire about the organisational characteristics of the intervention was developed specifically for this study and tailored to the elements of the intervention.

## Results

### Part 1

#### Availability of the intervention

**Figure 1:**
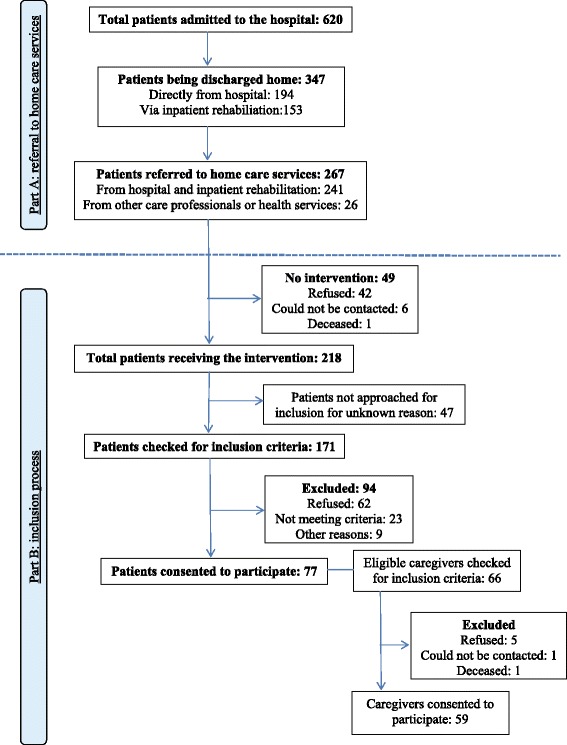
**Flow chart of (A) the referral of stroke patients to home care services and (B) the inclusion process.**

The first part of the process-evaluation focused on the discharge destination of stroke patients after hospitalisation or inpatient rehabilitation. Figure 1-part A shows that out of a total of 620 stroke patients, who were admitted to the stroke unit of the hospital during the inclusion period, 347 patients were discharged home, 241 of them being referred to home care services (69% of the total number of stroke patients). In addition, there were 26 patients who were referred to the home care services by other health care professionals, such as physiotherapists, GPs and nurse practitioners, who were left out of consideration for the evaluation about the availability of the intervention. The total of 267 patients that were referred to the home care services were offered the intervention, which was implemented as regular care in the Maastricht area at the beginning of the study period.

### Part 2

#### Participants and response

The second part of the evaluation concentrated on the intervention and Figure 1-part B provides an overview of the inclusion process of the trial. There were 218 patients who received the intervention, 77 patients of whom consented to participate in the evaluation. These included patients had 66 eligible caregivers, of whom 59 of them consent to participate. These patients and caregivers also participated in the effect evaluation [11].

The structured assessment forms of all 77 included patients were available. The SASC-19 was administered to 69 patients at T0 (90%), 59 at T6 (77%), 61 at T12 (79%) and 64 at T18 (83%). There were 13 patients who did not fill in the SASC-19 at the end of the study, for various reasons; four had died within the 18-month period, four had been admitted to a nursing home during the study, four had dropped out because of the intensity of the follow-up measurement, and one had severe dementia after 18 months. A total of 51 patients (66%) responded to the questionnaire about the evaluation of the care model. The remaining 26 patients did not return the questionnaire, 13 of them for the reasons mentioned above. The other 13 had various reasons for not responding: seven patients failed to respond after having been reminded by the researcher and six could not recall the care that they had received.

Fifty-four caregivers filled in the SASC-19 at T0 (92%), 38 at T6 (64%), 36 at T12 (61%) and 38 at T18 (64%). There were 21 caregivers who did not receive the SASC-19 at the end of the study period, for various reasons; eight caregivers dropped out for personal reasons (N = 5) or because of the intensity of the follow-up measurements (N = 3), for six caregivers the intervention was ended because the patients died during the study (N = 4) or were admitted to a nursing home (N = 2), three caregivers dropped out for unknown reasons, two caregivers died and two caregivers could not be reached. The questionnaire about the evaluation of the intervention was filled in by 29 caregivers (49%) at the 18-month follow-up. Thirty caregivers did not return the questionnaires, for various reasons, including the reasons mentioned above by the 21 patients: seven caregivers failed to respond after the reminder, and two caregivers could not recall the care that they had received. Demographic data and characteristics of patients and caregivers are shown in Table 1. All four SCCs returned the questionnaire about the organisational characteristics of the intervention that they provided.

**Table 1:** **Participants characteristics at baseline**

		**Patients**
Number		77
Age	(mean in years + SD)	72.5 (9.6)
Gender	(male/%)	41/53.2%
Time since stroke	(mean in months + SD)	1.8 (1.4)
Discharged from (N/%)	Hospital	52/67.5%
	Inpatient rehabilitation	25/32.5%
		**Caregivers**
Number		59
Age	(mean in years + SD)	61.5 (14.7)
Gender	(male/%)	18/30.5%
Relationship	Spouse	36/61.0%
	Child	17/28.8%
	Other	6/10.2%

N, number; SD, standard deviation.

### Performance of the intervention

Table 2 illustrates the number of home visits and the follow-up period. Forty-nine patients received fewer than the suggested five home visits. There were several reasons for this: in some cases the SCC considered that no further follow-up care was needed (N = 24), seven patients ended the intervention at their own initiative, three patients died, five patients were transferred to a home for the elderly, for eight patients the reasons are unknown and two patients received four home visits within the 18-month study period and were still receiving the intervention at the end of this period. Eight patients received more than five home visits, for the following reasons: one patient experienced more problems and needed more care, three patients had had a recurrent stroke and for four patients the reasons remained unknown. Thirty-one patients had the maximum follow-up period of 18 months, while 11 patients had a longer follow-up period. There was no relation between the follow-up period and the number of home visits (i.e. some patients had a shorter and more intensive follow-up period, while some patients had a longer and less intensive follow-up period). With regard to receiving the suggested home visit at the suggested time during the follow-up period, the first home visit was provided within one month for 66 patients. Subsequently, one out of four patients received the suggested home visits at the suggested time during the follow-up. With regard to the use of the validated measurement instruments, 74% of all identified problems were further explored by means of the validated measurements instruments. The problem domain of fatigue was further explored in 52% of the cases. Reasons for not further exploring the problem domains were unknown.

**Table 2:** **Overview of the home visits and follow-up period during the study period (N = 77)**

Home Visits (N = 5)		
Range (N)	1-7	
Average home visits (SD)	3.8 (1.5)	
One home visit	7	9%
Two home visits	10	13%
Three home visits	12	16%
Four home visits	20	26%
Five home visits	20	26%
Six or more home visits	8	10%
Follow-up period (18 months intended)	N	%
Range (months)	1-27	
Mean (months, SD)	13.3 (6.9)	
Maximum follow-up period of 1 month	6	8%
Maximum follow-up period of 3 months	5	7%
Maximum follow-up period of 6 months	9	12%
Maximum follow-up period of 12 months	15	19%
Maximum follow-up period of 18 months	31	40%
Follow-up period longer than 18 months	11	14%
Home visits and follow-up period in accordance with the protocol		
1^st^ home visit within 0-1 month	66	86%
2^nd^ home visit within 2-4 months	46	60%
3^rd^ home visit within 5-7 months	26	34%
4^th^ home visit within 11-13 months	22	29%
5^th^ home visit within 17-19 months	16	21%

N, number; SD, standard deviation.

### Follow-up care based on assessment

The SCCs administered the assessment tool during each home visit. A total of 1074 problems were identified during all assessments of all patients (N = 293). The stroke patients experienced an average of six problems at one to three months after discharge, a number which decreased to four problems after six months, increased to five after 12 months and decreased again to three after 18 months. Table 3 shows the main problems, experienced by the stroke patients and caregivers, with fatigue remaining the most important problem throughout the 18-month follow-up period.

**Table 3:** **The six main problems of patients and caregivers assessed at the predetermined follow-up home visits**

	**0-1 months (N = 66)**		**2-4 months (N = 53)**		**5-7 months (N = 40)**		**11-13 months (N = 32)**		**17-19 months (N = 17)**	
	**Problem**	%*	**Problem**	%*	**Problem**	%*	**Problem**	%*	**Problem**	%*
1	Fatigue	79	Fatigue	68	IADL	55	Fatigue	53	Fatigue	53
2	IADL	64	IADL	64	Fatigue	50	IADL	47	Cognition	47
3	Cognition	56	Cognition	57	Cognition	38	Cognition	44	IADL	47
4	Medical status	52	Social activity	49	Medical status	38	Communication	41	Communication	44
5	Communication	45	Communication	43	ADL	33	Social activity	38	Medical status	41
6	Social activity	44	Medical status	42	Social activity	28	Psycho-emotional	31	Psycho-emotional	24

*Percentage of patients who received care from the SCC and experienced this problem.

N, number; IADL, instrumental activities of daily life.

Based on all assessments performed, the SCCs provided supportive listening (N = 104), provided advice and information (N = 88), on aspects like lifestyle changes, coping with fatigue and rules about driving a car after a stroke, and initiated help for caregivers (N = 18). In addition to the care directly initiated by the hospital or inpatient rehabilitation service, they referred patients and caregivers to speech therapists (N = 11), occupational therapists (N = 9), physiotherapists (N = 8) and sometimes a dietician, social worker or psychologist (N = 28). They also arranged health care facilities (such as social alarm devices and personal transfer; N = 33), physical aids (such as wheel chair, adjusted shoes, mobility scooter; N = 24), home care (such as help with housekeeping; N = 20) and home adjustments (such as a stair lift, handrails in toilet and shower; N = 20). The SCCs initiated on average four to five follow-up care and referral options to each patient.

The SCCs had contacts with general practitioners 48 times, the most common reasons being referral of patients to other health care professionals for consultation or therapy, medical problems and use of medication. The SCCs also had contacts with the nurse practitioner at the hospital (N = 26), the neurologist (N = 15), physiotherapists (N = 10) and occupational therapists (N = 8) for information and advice. Other health care professionals they contacted were rehabilitation physicians (N = 4), psychologists (N = 4), cardiologists (N = 2), a speech therapist (N = 1) and home services (N = 1). The SSCs did not consult the multidisciplinary team during the follow-up period.

### Opinions of patients and caregivers

#### Patients

The average scores on the SASC-19 indicate that patients were satisfied with the intervention during the follow-up period (average scores ranging from 1.9 to 2.6) (Table 4). About 93% indicated that they had received all the help they needed during the 18-month period. The results also showed that 33% of the patients received insufficient information about financial resources and aids within the first few weeks after being discharged home.

**Table 4:** **Mean SASC-19 scores at the follow-up measurements**

**Patients**		**T0**	**T6**	**T12**	**T18**
SASC-19, part 1*	N	68	54	54	53
	Mean (SD)	2.4 (0.4)	2.5 (0.5)	2.5 (0.5)	2.6 (0.5)
	Score >1.5** (%)	100%	100%	100%	100%
SASC-19, part 2*	N	69	59	61	64
	Mean (SD)	1.9 (0.3)	1.9 (0.2)	1.9 (0.2)	2.0 (0.2)
	Score >1.5 (%)	91.3%	96.6%	95.1%	97.9%
Caregivers					
SASC-19, part 1*	N	47	33	32	34
	Mean (SD)	2.4 (0.5)	2.5 (0.5)	2.6 (0.5)	2.6 (0.5)
	Score >1.5** (%)	97.9%	100%	96.9%	94.1%
SASC-19, part 2*	N	54	38	36	38
	Mean (SD)	1.9 (0.4)	2.0 (0.4)	2.1 (0.3)	2.0 (0.4)
	Score >1.5** (%)	88.9%	94.7%	97.2%	97.4%

N, number; SD, standard deviation.

*Part 1: items relating to care by stroke care coordinator; part 2: items relating to care after hospitalisation or inpatient rehabilitation.

**Score of 0-1.5 total to moderate dissatisfaction; score of 1.6-3: moderate to total satisfaction.

With regard to the way the intervention was organised, 14 patients (30%) indicated that the first home visit should have taken place sooner after their discharge home (they received their first home visit within one to six weeks after being discharged). This was not related to whether patients had been discharged from the hospital (N = 7) or from inpatient rehabilitation (N = 7). About 8% of the patients would have preferred more home visits and 6% of the patients would have preferred a longer duration of the intervention. In addition, eight patients (16%) were of the opinion that the face-to-face contact with the SCC could be replaced by telephone contacts. All patients who filled in the questionnaire were satisfied with the duration of each home visit. With regard to improving their functioning in daily life, which was one of the major goals of the intervention, 65% of the patients said that the home visits and follow-up care they had received had improved their daily life functioning. They commented that they had received enough attention from the SCC and that the SCC gave useful advice and arranged effective follow-up care. Patients rated the care at an average of 8.6 out of 10 (i.e. very good).

#### Caregivers

Caregivers were satisfied with the care they received after the patient’s discharge during the follow-up period (average scores ranging from 1.9 to 2.6) (Table 4). They had received sufficient help from health care services (91%) and sufficient emotional support (78%) during the 18 months follow-up.

With regard to the way the intervention was organised, six caregivers (22%) indicated that the first home visit should have taken place sooner after the discharge. Five caregivers would have preferred more home visits within the first months after discharge (17%). About 68% of the caregivers reported that care would be inaccessible to them if it was provided at a health centre instead of their own home. Sixty-seven percent of the caregivers reported that the home visits they had received had improved their daily life functioning. The caregivers commented that the SCC had paid sufficient attention to them and that the SCC had discussed each problem satisfactorily. They rated the care at an average of 8.2 out of 10 (i.e. very good).

### Opinion of the stroke care coordinators

All SCCs (N = 4) preferred using the structured assessment procedure for each stroke patient and caregiver compared to their previous work approach, when they had used no structured assessment. They commented that the use of the assessment tool provided structure and an overall picture, and made the care provided during the home visits more efficient. The SCCs were generally satisfied with the frequency and timing of the home visits and the duration of the follow-up period. In several cases the frequency of the home visits and duration of follow-up depended on the patient’s individual situation, so the intervention was not performed in accordance with the suggested intervention. Patients with severe aphasia or cognitive impairments were more difficult to interview with the ACAS tool. The SCCs reported that they would have appreciated a more structured procedure to refer patients and caregivers to other follow-up care and stroke care professionals. They were dissatisfied about the lack of contact with general practitioners, whereas the collaboration with the other health care professionals was judged to be very easy and positive.

## Discussion

This study aimed to examine process-related factors that could have influenced the effectiveness of our intervention. Firstly, we assessed how many patients were referred to an SCC and found that 69% of the patients were referred to the home care services for the intervention after being discharged home. One third of the patients were not referred from the hospital or inpatient rehabilitation service to home care services to receive the intervention. These patients may have fully recovered from the stroke during hospitalisation or inpatient rehabilitation. The health care professionals may have considered that no further intervention was needed and discharged the patients home without further care, before the SCC had the opportunity to carry out a structured assessment of the patient’s needs. Health care professionals should therefore raise more public support for the referral of stroke patients to the home care services.

Secondly, we explored to what extent the intervention was performed in accordance with the protocol. In the course of the study, it appeared that the SCCs performed the follow-up care in a more flexible way and adapted the number of home visits and follow-up period, indicating that the intervention was only partially performed in accordance with the protocol. Forty-six percent of the patients and caregivers received home visits over a maximum period of 12 months, and 72% received less than the intended five home visits. The SCCs reported that they considered that some patients had no further need for follow-up care and that they ended the care before the intended 18-month follow-up period. In addition, the number of home visits and the timing of the home visits sometimes differed between patients (i.e. some patients had a shorter and more intensive follow-up period and some had a longer and less intensive follow-up period), suggesting that the intervention should perhaps be tailored to the patient’s individual situation, using our protocol as a guideline. The tailored intervention received by patients and caregivers could partially explain the limited effectiveness of the intervention [11], while the effectiveness was measured for the overall group and not measured for the patient’s individual situation.

Thirdly, we assessed the outcome of the assessment procedure and the follow-up care provided. The problems of fatigue, cognition, communication and IADL remained the most important problems during the follow-up period. The number of problems experienced by each stroke patient had decreased after 18 months, showing that patients experienced fewer problems and may have less need for follow-up care, as was indicated by the fewer home visits provided by the SCCs and the shorter follow-up period. The SCCs most commonly provided advice, information and supportive listening to patients and caregivers during the home visits. These results are comparable to the study results by Boter and colleagues (2004) showing that nurses who provided three telephone consults and one home visit to stroke patients most commonly provided supportive listening and information [21]. The SCCs in our study provided follow-up care for only one out of three of the problems identified. Based on the reporting of the SCCs, we could not derive what follow-up care was provided for what type of problem. Perhaps the initiated follow-up care by the SCC could have addressed more than one problem. It is also possible that not all of the initiated follow-up care was properly reported in the assessment forms of each patient. Relatively little follow-up care was initiated to resolve problems such as cognition and fatigue. It is possible that the SCCs were unfamiliar with effective interventions or referral options in the region. Our intervention may also have focused too much on the assessment of stroke-related problems rather than on follow-up interventions or referral of stroke patients, which could explain the limited effectiveness of the intervention [11]. Perhaps a more structured and systematically organised referral system is required to improve the continuity of follow-up care, as was suggested by the SCCs. Regarding the collaboration with other health care professionals, the SCCs were dissatisfied about the accessibility of and collaboration with the GPs. Several studies have also indicated that not only health care professionals but patients and caregivers as well prefer a more proactive role of their GP in providing follow-up care after being discharged home [22,23]. In addition, the SCCs never consulted the multidisciplinary team which was specifically organised as part of the intervention.

Fourthly, we explored the opinions of patients and caregivers about the intervention. Both groups were very satisfied with the care they had received after being discharged home. Finally, our evaluation of the opinion of the SCCs about the intervention showed that the SCCs were satisfied with the use of the ACAS tool during the home visits, which is in line with the results of a previous study by Murray and colleagues (2006) [24]. They evaluated the feasibility of a follow-up care intervention and found that health care professionals could work in a more consistent manner using a systematic assessment procedure. The SCCs would prefer a more structured referral system combined with the structured assessment procedure.

### Limitations

Our study had several weaknesses. First, there may have been a response bias due to socially desirable answers. We tried to minimise this bias by asking the patients and caregivers to fill in the questionnaire themselves and return it by post. Secondly, there were some missing data during the study period because of drop-out among patients and caregivers, but the reasons for drop-out were hardly related to the way the intervention was organised. Moreover, there was a large group of patients who were not asked to participate in the process evaluation, for unknown reasons. Thirdly, the data on the assessment and follow-up care were based on the clinical notes made by the SCCs, which may not have contained all relevant information. In addition, the SCCs’ clinical notes did not enable us to specifically indicate which follow-up care was initiated for what type of problem. Finally, we had changed the word ‘hospital’ in ‘hospital, nursing home or home care services’ in several items of the SASC-19. Although, we do not expect that this minor change will have affected the validity of the SASC-19, we have to consider this possibility.

## Conclusion

The study results show that the intervention was not always performed in accordance with the protocol, but the intervention was offered to patients in a more flexible way, and patients and caregivers were very satisfied with the follow-up care that they received. In addition, the health care professionals were satisfied about providing follow-up care by means of a structured assessment procedure. The results also suggest that the intervention should be tailored to each individual patient that is, adjusted to each patient’s needs.

The results of the trial showed that the intervention was effective in improving the levels of social activities [12], but perhaps more effects could have been found, if sufficient follow-up referral had been provided. We therefore have several recommendations for the way follow-up care should be organised, and for future research. First of all, we believe that the frequency and duration of the follow-up care should be tailored and not be strictly five home visits for a fixed period of 18 months. We also believe that an effective referral system to specific care related to the problems identified may be most important to improve the follow-up care for stroke patients. Therefore, we highly recommend the use of a structured assessment procedure combined with a structured referral system to guarantee the continuity of the follow-up care. In addition, we recommend that the health care professionals performing the assessment should be taught about effective intervention and referral options for problems such as cognition and fatigue [25,26]. The involvement of GPs should be integrated more fully into the follow-up care, because they are the primary health care professional of patients in the home situation for many years after the stroke. Our recommendations for future research are that the characteristics of patients who need long-term follow-up care should be studied to identify prognostic characteristics for follow-up care and the recording of follow-up care initiated should be registered in a more objective method, independent of the health care professional providing the care.
